# RETRACTED: Surface modification of TiO_2_ nanoparticles with biodegradable nanocellolose and synthesis of novel polyimide/cellulose/TiO_2_ membrane [J. Colloid Interface Sci. 491 (2017) 390–400]

**DOI:** 10.1016/j.jcis.2017.10.016

**Published:** 2018-01-15

**Authors:** Hashem Ahmadizadegan

**Affiliations:** Department of Chemistry, Isfahan University of Technology, Isfahan, Iran

This article has been retracted: please see Elsevier Policy on Article Withdrawal (https://www.elsevier.com/about/our-business/policies/article-withdrawal).

This article has been retracted at the request of the Editor-in-Chief as there is significant overlap with an earlier published article, without any reference to this paper. The article is: Surface modification of TiO_2_ nanoparticles with biodegradable nanocellolose and synthesis of novel polyimide/cellulose/TiO_2_ membrane, by Hashem Ahmadizadegan, Journal of Colloid And Interface Science, 491 (2017) 390–400, doi: https://doi.org/10.1016/j.jcis.2016.11.043.

